# Safety profiles of methylphenidate, amphetamine, and atomoxetine: analysis of spontaneous reports submitted to the food and drug administration adverse event reporting system

**DOI:** 10.3389/fphar.2023.1208456

**Published:** 2023-08-14

**Authors:** Wei Wei, Li Chen, Hui Zhou, Jinfeng Liu, Yue Zhang, Shiyu Feng, Yingtao Bai, Yanen Leng, En Chang, Liang Huang

**Affiliations:** ^1^ Department of Pharmacy, Evidence-Based Pharmacy Center, West China Second University Hospital, Chengdu, China; ^2^ Key Laboratory of Birth Defects and Related Diseases of Women and Children, Sichuan University, Ministry of Education, Chengdu, Sichuan, China; ^3^ Department of Pharmacy, People’s Hospital of Zhongjiang County, Deyang, China; ^4^ Department of Pediatrics, West China Second University Hospital, Chengdu, China; ^5^ Department of Pharmacy, Mianyang Orthopaedic Hospital, Mianyang, China

**Keywords:** methylphenidate, atomoxetine, amphetamine, FDA adverse events reporting system, dextroamphetamine, dexmethylphenidate, methamphetamine, lisdexamfetamine

## Abstract

**Background:** Methylphenidate, atomoxetine, and Amphetamine are the three most commonly used medications approved by the United States Food and Drug Administration (FDA) for the treatment of attention deficit/hyperactivity disorder (ADHD). However, a comprehensive analysis of their safety profiles across various age groups and genders in real-world contexts has yet to be conducted. In this study, a pharmacovigilance analysis was performed using the FDA Adverse Event Reporting System (FAERS) database to examine differences in adverse events between methylphenidate, atomoxetine, and Amphetamine.

**Methods:** From January 2014 to September 2022, FAERS reports listing “Methylphenidate,” “Dexmethylphenidate,” “Atomoxetine,” “Amphetamine,” “Lisdexamfetamine,” “Dextroamphetamine,” and “Methamphetamine” as primary suspects were analyzed after removing duplicate reports. We used the standardized Medical Dictionary for Regulatory Activities (MedDRA) query generalized search for adverse events at the preferred term level based on case reports. After filtering duplicate reports, disproportionality analysis was used to detect safety signals according to the proportional reporting ratio (PRR). In order to delve into potential safety concerns, we undertook a two-step analysis of the data. Initially, the data was segmented based on age cohorts: 0–5 years, 6–12 years, 13–18 years, and individuals aged ≥19 years. Following this, after partitioning the data into males and females within the 0–18 years age group, and similarly for those aged ≥19 years, further analysis was conducted.

**Results:** The pharmacovigilance analysis uncovered substantial safety signals in the standardized MedDRA queries. Methylphenidate was associated with dyskinesia (PRR = 21.15), myocardial infarction (PRR = 12.32), and hypertension (PRR = 8.95) in children aged 0–5, 6–12, and 13–18 years, respectively, as well as neonatal exposures via breast milk (PRR = 14.10) in adults aged ≥19 years. Atomoxetine was linked to hostility/aggression (PRR = 15.77), taste and smell disorders (PRR = 6.75), and hostility/aggression (PRR = 6.74) in children aged 0–5, 6–12, and 13–18 years, respectively, as well as hostility/aggression (PRR = 14.00) in adults aged ≥19 years. Amphetamine was associated with psychosis and psychotic disorders (PRR = 16.78), hostility/aggression (PRR = 4.39), and Other ischaemic heart disease (PRR = 10.77) in children aged 0–5 years, 6–12 years, and 13–18 years, respectively, and hostility/aggression in adults aged ≥19 years (PRR = 9.16). Significant and noteworthy adverse event signals were also identified at the preferred term level. Specifically, methylphenidate was associated with myocardial infarction, acute myocardial infarction, coronary artery dissection, electrocardiogram QT prolonged, growth retardation, self-destructive behavior, suicidal ideation, and completed suicide. Atomoxetine was linked to electrocardiogram QT prolonged, growth retardation, and tic. Amphetamine was recorded for coronary artery dissection, suicidal ideation, and completed suicide. It was observed that male patients, including both children and adults, showed a more significant and frequent occurrence of adverse events compared to females, particularly in terms of cardiac disorders. The intensity and quantity of adverse event signals were distinctly different between the two genders, with males having a higher number of signals. All detected safety signals were confirmed using signals obtained from the disproportionality analysis.

**Conclusion:** This pharmacovigilance analysis demonstrated significant variations in the safety profiles of methylphenidate, atomoxetine, and Amphetamine across different age groups and between different genders. Following an in-depth analysis of the FAERS database, we discerned prominent safety signals. Notably, the strength of the signals associated with coronary artery dissection induced by methylphenidate and amphetamine, as well as those related to suicide, demand particular attention. Consequently, it remains imperative to persist in monitoring these medications, assessing the associated risks, and carrying out comparative studies particularly geared towards ADHD drugs.

## 1 Introduction

Attention deficit/hyperactivity disorder (ADHD) is a neurodevelopmental disorder that manifests during childhood. It is characterized by symptoms such as hyperactivity, impulsivity, and inattention. These symptoms influence a child’s cognitive function, academic performance, behavior, emotional wellbeing, and social skills (Wolraich et al., 2019). ADHD develops in approximately 9%–15% of school-aged children, rendering it one of the most common disorders in childhood (Merikangas et al., 2010; Wolraich et al., 2014; Rowland et al., 2015; Zablotsky et al., 2019). Research suggests that almost 90% of children with ADHD eventually require pharmacological treatment (Stein, 2008; Danielson et al., 2018). Furthermore, approximately 60% of patients continue to exhibit symptoms into adulthood, leading to significant psychological, occupational, and social impairments throughout their lives (Kooij et al., 2010). Psychostimulants, including amphetamines and methylphenidate, are first-line pharmacotherapies for individuals with ADHD. Atomoxetine is the first non-stimulant medication approved by the United States Food and Drug Administration (FDA) for the treatment of ADHD. It is a selective norepinephrine reuptake inhibitor that can be employed to treat ADHD in children, adolescents, and adults, offering an alternative to methylphenidate (Kendall et al., 2008; Wolraich et al., 2011). In addition, both methylphenidate and atomoxetine have been approved by the FDA for the treatment of narcolepsy, while methamphetamine has been approved for the short-term treatment of exogenous obesity. ADHD is a chronic condition necessitating long-term medication. Therefore, the tolerability and safety of therapeutic interventions for ADHD are of paramount concern to regulators, healthcare providers, and caregivers alike (Cortese et al., 2013). Despite the demonstrated efficacy and good tolerability of ADHD medications, potential adverse reactions, particularly those involving cardiovascular and psychiatric aspects, remain a substantive issue (Clavenna and Bonati, 2017). Studies have already shown that ADHD medications may work differently for males and females. However, there has not been a comprehensive study on the gender-based differences in the negative side effects of these medications yet (Kok et al., 2020). The use of methylphenidate and other medications for ADHD continues to increase rapidly in numerous countries, underscoring the importance of issuing appropriate warnings regarding potential adverse effects.

This study performs a pharmacovigilance analysis using the FAERS database. Initially, Patients are initially categorized into various age groups for analysis. The subsequent analysis then separates these patients into two specific groups: males and females aged 0–18 years, and males and females aged ≥19 years. The primary objective is to examine the discrepancies in adverse events among patients of different age groups and genders who use methylphenidate, atomoxetine, and amphetamine in real-world situations. The study emphasizes the crucial need for continuous monitoring, risk assessment, and further comparative research.

## 2 Materials and methods

### 2.1 Data sources

This is a retrospective study utilizing the FAERS database, which gathers voluntary reports of adverse reactions and medication errors from healthcare professionals, patients, and pharmaceutical manufacturers worldwide (Dagenais et al., 2018). This publicly accessible database enables the analysis of extensive data to identify safety signals. The ability of FAERS to detect early safety concerns has been previously documented, especially for newly approved medications (Fukazawa et al., 2018) and rare adverse events (AEs) (Harpaz et al., 2013). Data for this study were retrieved from the public release of the FAERS database, which adheres to the international safety reporting guidance issued by the International Conference on Harmonisation (ICH E2B). OpenVigil FDA (Böhm et al., 2021), a pharmacovigilance tool, was employed to extract data from the FAERS database. The classification and standardization of AEs in the FAERS data are based on the Medical Dictionary for Regulatory Activities (MedDRA) (Brown et al., 1999). In the FAERS database, each report is coded using preferred terms (PTs) from MedDRA terminology; a given PT can be assigned to one or more High-level Terms, High-level Group Terms, and System Organ Class levels within MedDRA. Furthermore, different PTs can be amalgamated to define a specific clinical syndrome using an algorithmic approach termed standardized MedDRA queries. Definitions provided by MedDRA were utilized in this study.

### 2.2 Data processing and AE signal detection

From January 2014 to September 2022, FAERS reports listing “Methylphenidate,” “Dexmethylphenidate,” “Atomoxetine,” “Amphetamine,” “Dextroamphetamine,” “Lisdexamfetamine,” and “Methamphetamine” as primary suspects were analyzed after removing duplicate reports (i.e., with the same identifier number). Two researchers used standardized MedDRA query and PT to categorize related AEs, and extracted patient and drug information from the reports. The data extracted included the gender, age, drug name, indication, event, outcome, date received, and so on. Disproportionality analyses were conducted using OpenVigil 2.1. In the “Data Presentation and Statistics Box” of OpenVigil 2.1, the proportional reporting ratio (PRR) was calculated to assess the adverse effects of methylphenidate, atomoxetine, and amphetamine. Table 1 illustrates the methodology employed for the calculation of the Proportional Reporting Ratio (PRR). A higher PRR suggests a stronger association; for example, a PRR of 2 indicates that the AE occurs twice as frequently in drug users compared with the background population. According to the criteria established by Evans et al. (2001), a positive signal of disproportionality was defined as a PRR ≥ 2, a chi-squared value ≥4, and at least three cases. The data was first segmented based on age cohorts: 0–5 years, 6–12 years, 13–18 years, and individuals aged ≥19 years. It was then further partitioned into males and females within the 0–18 years and ≥19 years age groups for a more detailed analysis.

**TABLE 1:** PRR algorithm used for signal detection.

	Adverse events of interest	All other adverse events of interest	Total
Drug of interest	a	b	a + b
All other drugs of interest	c	d	c + d
Total	a + c	b + d	a + b + c + d

PRR = [a/(a + b)]/[c/(c + d)], χ2 = [(ad-bc)^2^](a + b + c + d)/[(a + c) (b + d) (a + b) (c + d)]. Abbreviations: PRR, proportional reporting ratio.

## 3 Results

### 3.1 Descriptive analysis

**FIGURE 1:**
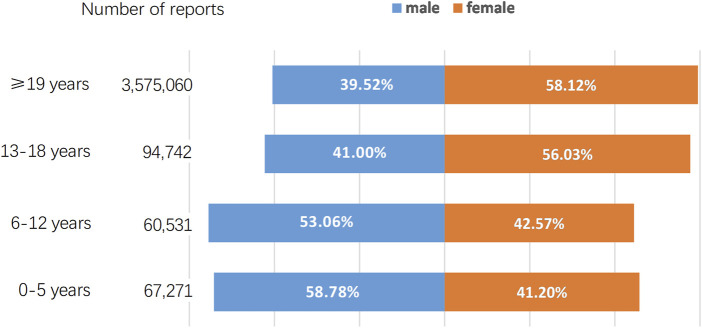
Proportional gender distribution of AE reports across various age groups.

As of September 2022, the FAERS database had received a total of 3,797,604 AE reports. A breakdown by gender reveals that males contributed 1,516,511 (39.93%) of these AE reports while females accounted for a higher proportion with 2,184,508 (57.52%) reports. The distribution of AE reports across various age groups, along with the percentage representation of both genders within each age group, is depicted in Figure 1. We further retrieved a total of 37,046 AE reports, including 15,073 reports for methylphenidate and dexmethylphenidate, 5,920 reports for atomoxetine, and 16,053 reports for amphetamine, dextroamphetamine, methamphetamine, and lisdexamfetamine. Table 2 describes the characteristics of AE reports submitted for these drugs. Consistent with the epidemiology of ADHD, the majority of reported patients were male (Xu et al., 2018). However, among amphetamine users, females accounted for 53.77%, surpassing male patients. Among methylphenidate and atomoxetine users, those aged ≤18 years accounted for 54.46% and 51.28% of cases, respectively. In contrast, in the population using amphetamines, only 18.42% of patients were 18 years old or younger.

**TABLE 2:** Clinical characteristics of patients from the FAERS database.

Characteristic	Number of reports, n (%)					
	Methylphenidate		Atomoxetine		Amphetamine	
Gender						
Male	9,333	(61.92)	3,372	(56.96)	6,969	(43.41)
Female	5,458	(36.21)	2,375	(40.12)	8,631	(53.77)
Unknown	282	(1.87)	173	(2.92)	453	(2.82)
Age (years)						
0–5	851	(5.65)	156	(2.64)	232	(1.45)
6–12	4,938	(32.76)	1,781	(30.08)	1,464	(9.11)
13–18	2,420	(16.06)	1,099	(18.56)	1,261	(7.86)
≥19	6,864	(45.54)	2,884	(48.72)	13,096	(81.58)
Year						
2022 (q1–q3)	1,763	(11.7)	472	(7.97)	2,817	(17.55)
2021	1,989	(13.2)	413	(6.98)	2,722	(16.96)
2020	1,208	(8.01)	199	(3.36)	1,894	(11.80)
2019	1,608	(10.67)	268	(4.53)	1,777	(11.07)
2018	2,214	(14.69)	242	(4.09)	2,095	(13.05)
2017	2,000	(13.27)	243	(4.1)	1,633	(10.17)
2016	1,539	(10.21)	394	(6.66)	1,276	(7.95)
2015	1,729	(11.47)	3,360	(56.76)	1,184	(7.38)
2014	1,023	(6.79)	329	(5.56)	655	(4.08)
Indication						
Attention deficit/hyperactivity disorder	4,498	(29.84)	1,953	(32.99)	4,018	(25.03)
Disturbance in attention	111	(0.74)	26	(0.44)	41	(0.26)
Narcolepsy	166	(1.10)	-		120	(0.75)
Autism spectrum disorder	121	(0.80)	16	(0.27)	12	(0.07)
Binge eating	-		-		89	(5.54)
Outcome						
Hospitalization	3,477	(23.07)	544	(9.19)	1,136	(7.08)
Death	965	(6.40)	112	(1.89)	1,433	(8.93)
Life-Threatening	700	(46.44)	184	(3.11)	361	(2.25)
Congenital Anomaly	263	(1.74)	20	(0.34)	10	(0.06)
Disability	301	(2.00)	62	(1.05)	619	(3.86)

Abbreviations: FAERS, United States food and drug administration adverse event reporting system; q1, quarter 1; q3, quarter 3.

### 3.2 Signal of standardized MedDRA queries

**FIGURE 2:**
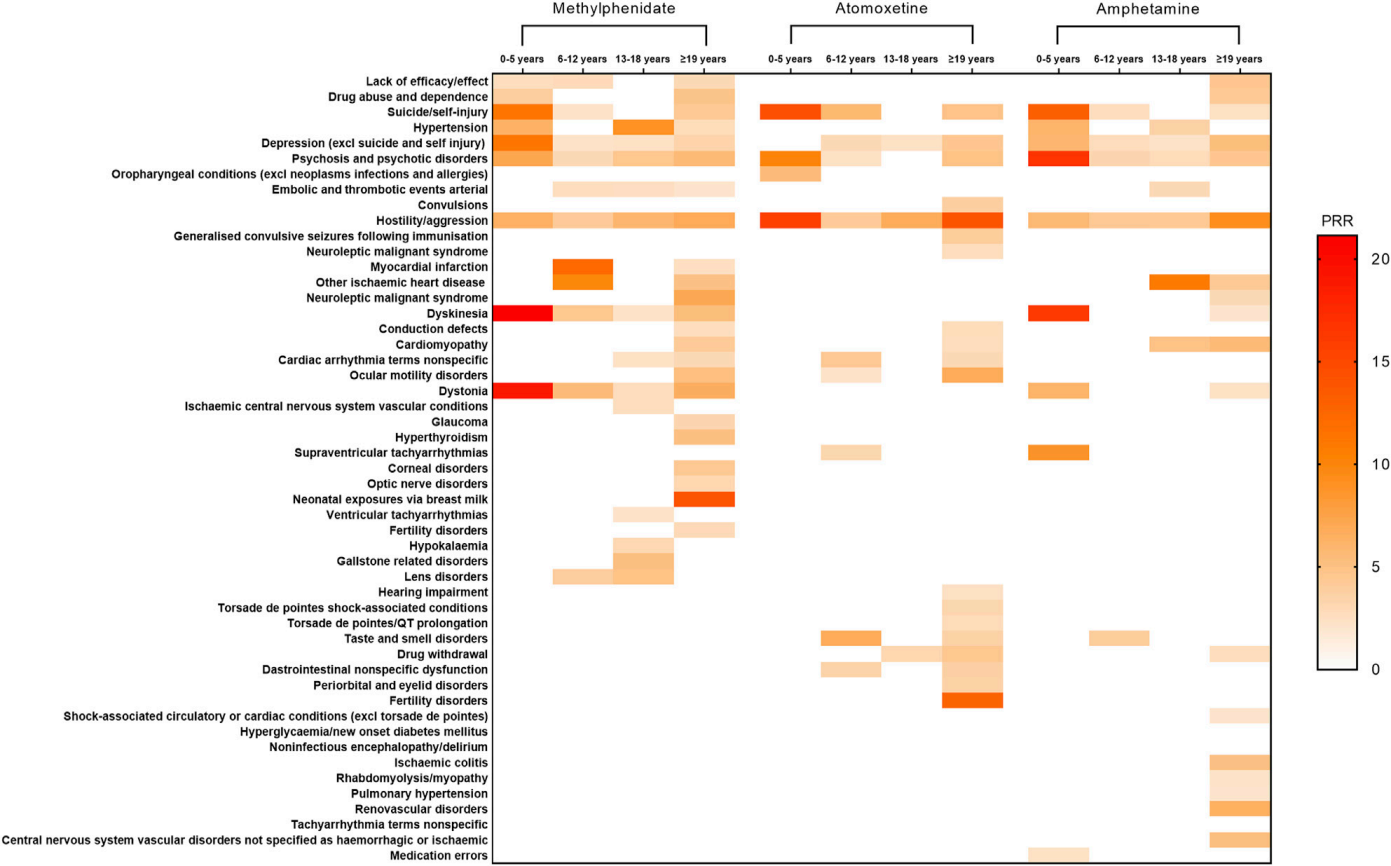
Positive signal distribution for methylphenidate, atomoxetine, and amphetamine using standardized MedDRA queries. Abbreviations: excl, excluding; MedDRA, Medical Dictionary for Regulatory Activiti; PRR, Proportional Reporting Ratio.

In this study, standardized MedDRA query searches were conducted for methylphenidate, atomoxetine, and amphetamine across different age groups. Moreover, signal detection was performed to comprehensively identify specific clinical cases with AEs related to these three drugs. Among methylphenidate users, the strongest signals for patients aged 0–5 years were linked to dyskinesia (PRR = 21.15), followed by dystonia (PRR = 19.13) and suicide/self-injury (PRR = 11.20). For those aged 6–12 years, the strongest signals were obtained for myocardial infarction (PRR = 12.31), followed by other ischemic heart diseases (PRR = 9.84) and dystonia (PRR = 5.48). For patients aged 13–18 years, the strongest signals were recorded for hypertension (PRR = 8.95), followed by hostility/aggression (PRR = 5.90) and gallstone-related disorders (PRR = 5.10). For those aged ≥19 years, the strongest signals were detected for neonatal exposures via breast milk (PRR = 14.1), followed by neuroleptic malignant syndrome (PRR = 7.07) and dystonia (PRR = 6.50). Among Atomoxetine users, the strongest signals for patients aged 0–5 years were obtained for hostility/aggression (PRR = 15.77), followed by suicide/self-injury (PRR = 14.45) and psychosis and psychotic disorders (PRR = 10.12). For those aged 6–12 years, the strongest signals were linked to suicide/self-injury (PRR = 5.73), followed by non-specific cardiac arrhythmia terms (PRR = 4.38) and hostility/aggression (PRR = 4.16). For patients aged 13–18 years, the strongest signal was detected for hostility/aggression (PRR = 6.74). For those aged ≥19 years, the strongest signals were recorded for hostility/aggression (PRR = 14.00), followed by fertility disorders (PRR = 12.73) and ocular motility disorders (PRR = 6.76). Among amphetamine users, the strongest signals for patients aged 0–5 years were linked to psychosis and psychotic disorders (PRR = 16.78), followed by dyskinesia (PRR = 16.00) and suicide/self-injury (PRR = 13.17). For those aged 6–12 years, the strongest signals were obtained for hostility/aggression (PRR = 4.39), followed by taste and smell disorders (PRR = 4.03) and psychosis and psychotic disorders (PRR = 3.19). For patients aged 13–18 years, the strongest signals were recorded for other ischaemic heart disease (PRR = 10.77), followed by cardiomyopathy (PRR = 4.87) and embolic and hostility/aggression (PRR = 4.32). For those aged ≥19 years, the strongest signals were detected for hostility/aggression (PRR = 9.16), followed by renovascular disorders (PRR = 6.35) and cardiomyopathy (PRR = 5.50). Detailed results are provided in Figure 2.

**FIGURE 3:**
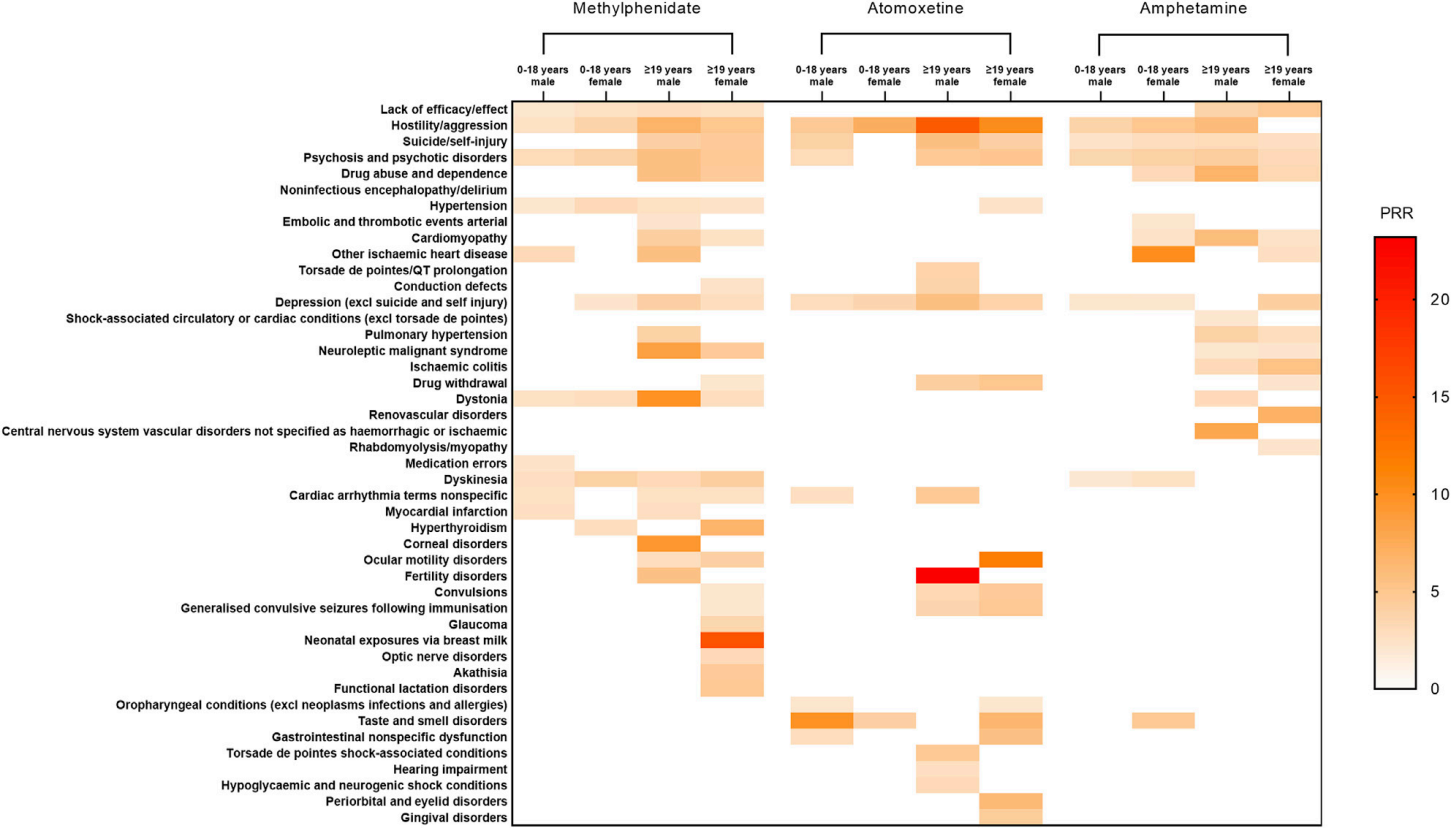
Positive signal distribution for methylphenidate, atomoxetine, and amphetamine using standardized MedDRA queries. Abbreviations: excl, excluding; MedDRA, Medical Dictionary for Regulatory Activiti; PRR, Proportional Reporting Ratio.

Furthermore, we stratified patients using these drugs based on gender. Among male methylphenidate users aged 0–18 years, the strongest signals were linked to other ischaemic heart disease (PRR = 3.17). For female patients within the same age range, the strongest signals were associated with dyskinesia (PRR = 3.99). Among male patients aged ≥19 years, the strongest signals were linked to dystonia (PRR = 9.78). In contrast, for females in the same age group, the strongest signals were detected for neonatal exposures via breast milk (PRR = 15.55). Among Atomoxetine users, the strongest signals for male patients aged 0–18 years were linked to taste and smell disorders (PRR = 9.76). For female patients within this age group, the strongest signals were associated with hostility/aggression (PRR = 7.18). For male patients aged ≥19 years, the strongest signals were linked to fertility disorders (PRR = 23.20). For females of the same age group, the strongest signals were recorded for ocular motility disorders (PRR = 11.66). Among amphetamine users, the strongest signals for male patients aged 0–18 years were associated with psychosis and hostility/aggression (PRR = 3.78). For females within this age group, the strongest signals were linked to other ischaemic heart disease (PRR = 10.08). For male patients aged ≥19 years, the strongest signals were detected for central nervous system vascular disorders not specified as haemorrhagic or ischaemic (PRR = 7.84). For females in the same age group, the strongest signals were recorded for renovascular disorders (PRR = 6.80). Detailed results are provided in Figure 3.

### 3.3 Signal of PTs

**FIGURE 4:**
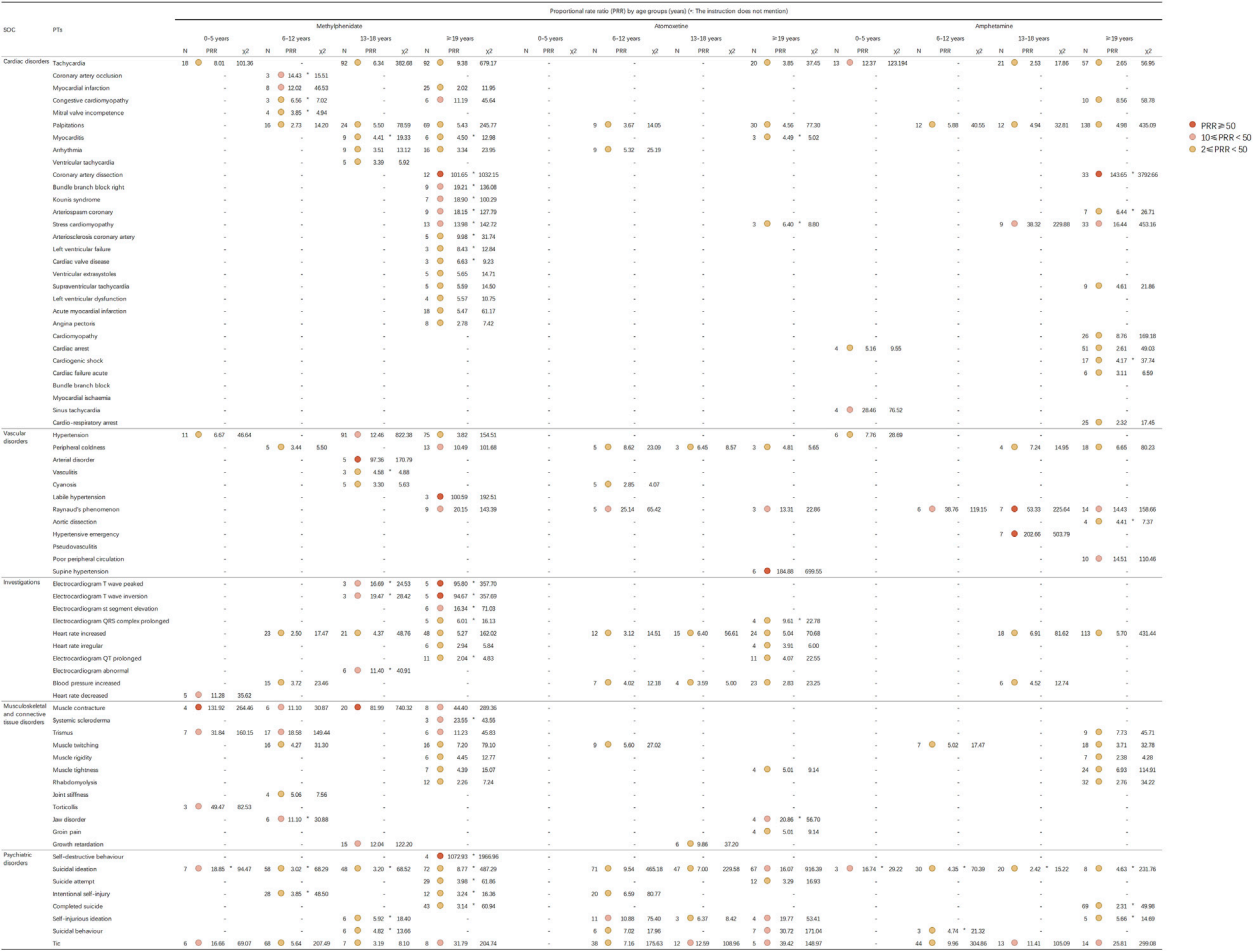
Signal strength for methylphenidate, atomoxetine, and amphetamine based on the PT level in FAERS. Abbreviations: FAERS, United States Food and Drug Administration Adverse Event Reporting System; PT, preferred term; SOC, System Organ Class.

**FIGURE 5:**
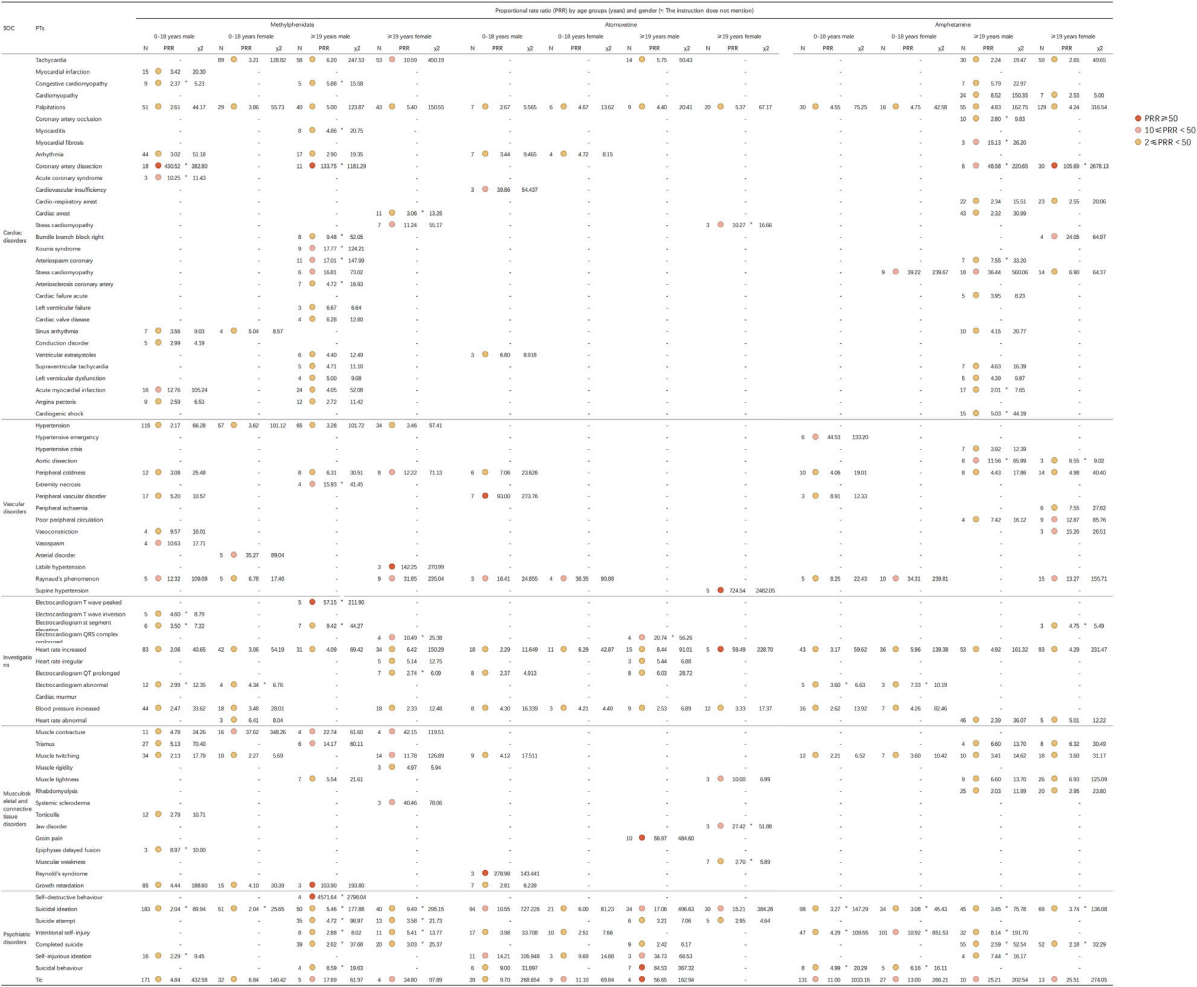
Signal strength for methylphenidate, atomoxetine, and amphetamine based on the PT level in FAERS. Abbreviations: FAERS, United States Food and Drug Administration Adverse Event Reporting System; PT, preferred term; SOC, System Organ Class.

In adherence to the latest guidelines (Wolraich et al., 2019), and considering our practical clinical experiences as well as concerns and anxieties of ADHD patients and their families encountered in our pharmaceutical outpatient department, We further detected PT signals and, in combination with FAERS data and literature review analysis, Firstly, we grouped by age and identified 72 PTs (involved in five System Organ Classes) for further exploration. We then stratified by gender and again identified 79 PTs (involved in five System Organ Classes) for further exploration. These System Organ Classes included cardiac disorders, vascular and lymphatic disorders, various examinations, musculoskeletal and connective tissue diseases, and psychiatric disorders. Figure 4 illustrates the adverse reaction signals of the three drugs by different age groups, while Figure 5 demonstrates the adverse reaction signals by different genders among both child and adult patients.

## 4 Discussion

In this study, we performed a pharmacovigilance analysis using the FDA Adverse Event Reporting System (FAERS) database to examine the differences in adverse events between methylphenidate and, atomoxetine and amphetamine. The results of our analysis unveiled the well-established adverse reactions listed on the drug labels, as well as the emergence of previously unreported and rare adverse reaction signals. Additionally, the medical community is growing increasingly aware of the influence of gender on therapeutic outcomes, with females increasingly seen as a risk factor for clinically relevant ADR (Franconi and Campesi, 2014; Anderson et al., 2018). Therefore, we also further investigated the differences in adverse reaction signals between male and female patients. This underscores the importance of ongoing pharmacovigilance in detecting and monitoring potential safety concerns associated with these medications.

According to our study, we note considerable differences in the manifestation of adverse reaction signals of these drugs across different ages and genders. Individual variations in physiological responses tend to increase with age as it influences structural and functional changes in organs. These alterations can impact how drugs are absorbed and cleared within the body, leading to changes in pharmacokinetics and drug sensitivity (Mangoni and Jackson, 2004). Additionally, since each ADHD medication exhibits some degree of gender-based efficacy (Kok et al., 2020), physiological differences between males and females could potentially lead to variations in adverse reactions. Furthermore, a study by Holm et al. (2017) points out that spontaneous reports of adverse reactions are influenced by age and gender, all of which could contribute to the observed differences in adverse reaction signals among different ages and genders.

### 4.1 Cardiac disorders, vascular disorders, and investigations

In the present study, we identified several adverse reaction signals related to the heart rate and blood pressure for both methylphenidate and atomoxetine across all age groups, as illustrated in Figure 4. Clinicians, patients, parents, and the general public have expressed significant concern regarding the cardiovascular safety of medications for ADHD (Kratochvil, 2012). Initial apprehensions regarding the cardiovascular safety of methylphenidate emerged in 1958 (Maxwell et al., 1958). By 1976, researchers discovered that treatment with methylphenidate substantially elevated the blood pressure and heart rate (Ballard et al., 1976). In 2012, it was revealed that children with ADHD exhibit autonomic dysfunction (Buchhorn et al., 2012). Treatment with methylphenidate and atomoxetine may further exacerbate the cardiovascular risk. Lamberti et al. (2015) observed that the average heart rate in children receiving methylphenidate increased from 80.5 ± 15.5 bpm to 87.7 ± 18.8 bpm; however, there were no significant changes detected in electrocardiogram parameters. To investigate the discrepancies in adverse reactions across different age groups, we analyzed the differences in adverse reaction signals among various age groups for the three medications. As studies have demonstrated physiological differences between males and females, with distinct gender differences in the clinical manifestation of cardiovascular diseases (Stolarz and Rusch, 2015). Hence, we further examined the differences in adverse reaction signals between males and females. The present analysis identified hypertension signals in patients of all age groups treated with methylphenidate and atomoxetine. Firstly, we detected adverse reaction signals affecting the heart rate in patients treated with methylphenidate, amphetamine and atomoxetine (except in those aged 0–5 years). Subsequently, through stratified analysis by gender, we found no significant differences between males and females. In general, methylphenidate and amphetamine manifested more pronounced adverse effect signals compared to atomoxetine. Consequently, we recommend that patients undergoing treatment with methylphenidate, amphetamine, or atomoxetine have their heart rate and blood pressure routinely monitored throughout the course of therapy. Despite the established efficacy, favorable safety profile, and extensive utilization history, lingering apprehensions remain regarding the likelihood of infrequent, yet severe, cardiovascular AEs linked to pharmacological interventions for ADHD. Of note, in patients aged ≥19 years, we identified signals of electrocardiogram QT prolongation as an adverse reaction associated with both methylphenidate (PRR = 2.04) and atomoxetine (PRR = 4.07). Through stratified analysis by gender, we found that signals of electrocardiogram QT prolongation were present in female patients aged ≥19 years who were administered methylphenidate (PRR = 2.74). For atomoxetine, these signals were detected in male patients, specifically in those aged 0–18 years (PRR = 2.37) and those aged ≥19 years (PRR = 6.03). Drug-induced fatalities predominantly stem from torsades des pointes, a potentially lethal polymorphic ventricular tachycardia frequently correlated with prolonged QT intervals. Given that the QT interval diminishes as the heart rate increases, it is customarily adjusted for heart rate (QTc). Drug-induced QT/QTc prolongation and torsades des pointes represent relatively uncommon adverse reactions to medications for ADHD commonly employed in clinical settings (Roden, 2004). A meta-analysis conducted by Martinez-Raga et al. (2013) posited that, when administered at therapeutic dosages, medications for ADHD are not linked to a heightened risk of cardiac incidents or other grave cardiovascular complications (inclusive of QTc prolongation) in pediatric, adolescent, or adult populations. Nevertheless, utmost prudence is warranted when contemplating the prescription of methylphenidate or atomoxetine for patients with ADHD of any age who present with personal or familial histories of cardiovascular disorders or other predisposing factors to the occurrence of cardiovascular events. Increased vigilance is necessitated when concurrently prescribing medications associated with the risk of cardiac AEs (Martinez-Raga et al., 2013).

Furthermore, it is important to highlight that, in patients aged ≥19 years using methylphenidate, we identified statistically significant adverse reaction signals for coronary artery dissection (PRR = 101.65), acute myocardial infarction (PRR = 5.47), myocardial infarction (PRR = 2.02), and electrocardiogram ST elevation (PRR = 15.58). In those aged 6–12 years, an adverse analysis signal for myocardial infarction was detected (PRR = 12.02). Among the users of amphetamines aged ≥19 years, we likewise identified an adverse reaction signal for coronary artery dissection (PRR = 143.65). Notably, through stratified analysis by gender, we found that signals of coronary artery dissection as an adverse reaction were exclusively present in male patients using methylphenidate, specifically in those aged 0–18 years (PRR = 430.52) and those aged ≥19 years (PRR = 133.75). Meanwhile, in patients aged ≥19 years using amphetamines, we detected signals of coronary artery dissection in both male and female patients, specifically in males aged ≥19 years (PRR = 48.58) and females of the same age group (PRR = 105.69). Coronary artery dissection is a major cause of acute myocardial infarction (Kim, 2020), and spontaneous coronary artery dissection is a rare, yet potentially severe, condition (Liang et al., 2018). A meta-analysis focusing on five studies with >43,000 children and adolescents did not find significant differences in adverse cardiac events between methylphenidate and atomoxetine. Similarly, a meta-analysis of three studies involving 775 adults did not reveal significant differences in adverse cardiac events between methylphenidate and placebo (Stammschulte et al., 2022). Nonetheless, AE reports from Canada and Germany (Wonnacott and Berringer, 2016; Stammschulte et al., 2022), including cases of acute myocardial infarction and coronary artery dissection, have raised concerns regarding the safety of these medications (Anders and Sharfstein, 2006). Furthermore, through a PubMed search, we discovered several case reports of amphetamine users experiencing coronary artery dissection, all suspected to be caused by the use of amphetamines. The present findings further emphasize the need for enhanced vigilance concerning the occurrence of severe cardiovascular AEs in patients using methylphenidate or amphetamine. Although the underlying mechanism remains to be clarified, the risk of myocardial infarction may be attributable to the cardiopressor dopaminergic/noradrenergic effects of psycho-stimulant drugs like amphetamine and methylphenidate, leading to increased heart rate and blood pressure (Volkow et al., 2003; Purper-Ouakil et al., 2011).

### 4.2 Musculoskeletal and connective tissue disorders

In patients aged 13–18 years, we identified adverse reaction signals associated with growth retardation for both methylphenidate (PRR = 12.04) and atomoxetine (PRR = 9.86). Further stratified by gender, we found that in atomoxetine users, growth retardation was only detected in male patients aged 0–18 years (PRR = 2.81). For those using methylphenidate, we found signals of growth retardation in both male and female patients aged 0–18 years, with PRR values of 4.44 and 4.10 respectively. The signal strength was approximately the same, but there were 85 reported cases in males, significantly more than the 15 cases reported in females. The potential impact of medications for ADHD on growth and development has long been a matter of concern. Conclusions from existing research remain contentious. The study conducted by Swanson et al. (2007) suggested that the central nervous system stimulant methylphenidate may impede growth and development. Their investigation examining the influence of the non-stimulant atomoxetine on growth and development in the treatment of ADHD, spanning a period >5 years, revealed that the effects of atomoxetine on the height and weight of children were transient, with a gradual rebound and recovery as treatment progressed. Longitudinal studies suggested that, during the initial 3 years of methylphenidate usage, height growth was impaired by 1 cm annually, representing a clinically significant reduction (Poulton, 2005). Some evidence indicates that these effects may wane over time, leaving the ultimate adult height unaffected by prior exposure to methylphenidate (Kramer et al., 2000; Faraone et al., 2008; Biederman et al., 2010; Peyre et al., 2013). Moreover, other researchers have reported that alterations in height or weight could be innate manifestations of ADHD rather than consequences of medication (Spencer et al., 1992; Swanson et al., 2007; Hanć and Cieślik, 2008). The possible influence of medications for ADHD on the growth and development of children and adolescents may stem from several factors. Several neurobiological mechanisms could potentially lead to the expected growth defects associated with methylphenidate. These may include the drug’s impact on liver and/or central nervous system growth factors, as well as its direct effect on cartilage. Dysregulation of molecular receptors involved in growth systems could explain the short-term effects of the drug. On the other hand, receptor adaptation over time may be the basis for tolerance to growth suppression and catch-up or compensatory growth after discontinuation of the stimulant (Cortese et al., 2013). As for atomoxetine, a meta-analysis of seven double-blind/placebo-controlled studies and six open-label studies found that the actual average weight and height at 24 months were 2.5 kg and 2.7 cm lower, respectively, than expected based on baseline weight and height percentiles (Kratochvil et al., 2006). However, the mechanism behind this occurrence still requires further investigation. Consequently, we advise that patients (especially adolescents) receiving methylphenidate and atomoxetine should continuously monitor their height and weight before and during treatment to evaluate their growth and development status.

### 4.3 Psychiatric disorders

The FDA has issued a black box warning for atomoxetine due to the potential elevation of suicidal ideation risk in children, urging clinicians to meticulously assess the risk-benefit ratio when prescribing this drug. An Italian investigation involved 2,239 patients with ADHD aged <18 years who were treated with either methylphenidate (1,268 cases, 56.7%) or atomoxetine (971 cases, 43.3%). The results revealed that all seven reported instances of suicidal ideation, self-harm, or related symptoms during treatment were observed in patients receiving atomoxetine, indicating an associated risk (Capuano et al., 2014). This finding aligns with our results, as we identified relevant signals in patients aged 6–12, 13–18, and ≥19 years using atomoxetine. In contrast, it is proposed that the stimulant medication methylphenidate or amphetamine exerts positive effects in mitigating the risk of suicide. A comprehensive review analyzing the influence of medication for ADHD on suicide-related behavior concluded that, unlike the non-stimulant atomoxetine, treatment with a stimulant significantly decreased suicidal intent in patients with ADHD of all ages (overall odds ratio = 0.72); notably, longer-term treatment with medication was correlated with a reduction in risk (Chang et al., 2020). Researchers suggested that stimulant therapy might lower the risk of suicidal behavior in patients with ADHD by ameliorating core symptoms, enhancing executive function, and diminishing the incidence of comorbidities (e.g., depression and substance abuse) over extended treatment periods (Öhlund et al., 2020). Nonetheless, we also detected signals of adverse reactions associated with suicide in patients using methylphenidate. The most prominent signals were self-destructive behavior in the ≥19 years age group (PRR = 1072.93), followed by completed suicide (PRR = 60.94), intentional self-harm (PRR = 16.36), and suicidal ideation detected across all age groups (0–5 years: PRR = 18.85; 6–12 years PRR = 3.02; 13–18 years: PRR = 3.2; ≥19 years: PRR = 8.77). In users of amphetamine, we also identified adverse reaction signals related to suicide across all age groups. Further stratification by gender revealed that the signal strength was generally stronger in male patients than in female patients. Instances of suicidal ideation resulting from the use of methylphenidate in the treatment of ADHD have been previously reported in the literature (Fettahoglu et al., 2009). Some reports have posited that suicidal ideation may arise from impulsivity as an inherent aspect of ADHD or potentially be a consequence of depressive moods induced by the use of methylphenidate. A case report from India documented two cases of suicidal ideation in male children initiating treatment with methylphenidate for ADHD (Arun and Sahni, 2014). The investigators contended that suicidal ideation occurred as a side effect of methylphenidate. Moreover, a Dutch cohort study indicated an increased risk of attempted suicide in adults aged <40 years following the commencement of treatment with methylphenidate (Tobaiqy et al., 2011; Stricker et al., 2022). The mechanism underlying the methylphenidate-induced risk of suicide remains unclear. For users of amphetamine, the mortality rate of individuals with stimulant use disorders, such as methamphetamine, is five times that of the general population, with suicide being one of the main causes of death. The reasons for suicide could be due to the direct impact of amphetamine, the adverse effects of psychosomatic comorbidities, or social factors. The rates of suicide and accidental deaths in males are significantly higher than those in females (Lee et al., 2021). Considering the present research findings, clinicians should closely monitor patients for the potential development of adverse reactions related to suicidal ideation when prescribing methylphenidate or amphetamine. Ensuring that the parents and teachers of patients receive education on potential adverse reactions associated with methylphenidate or amphetamine is of equal importance.

Unexpectedly, we detected signals related to Psychosis and psychotic disorders, as well as Suicide/self-injury as Adverse Drug Events (ADEs) in the age group of 0–5 years. The pathophysiology, vulnerability, and physical development of children diverge substantially from adults in various ways. Age differences often alter a child’s reaction to psychotropic drugs (Safer, 2011), with children, especially pre-schoolers, being particularly susceptible to stimulant-related ADEs. However, identifying adverse reactions in pediatrics is challenging, as many of the available tools are ill-suited for pediatric use (Bracken et al., 2018). Reporting adverse reactions in children poses a greater challenge than in adults, as it typically involves parents as critical intermediaries, and children may not be as capable of describing their symptoms as adults are (Blake et al., 2014). Despite Methylphenidate being recommended as the first-line treatment for pre-school children (Wolraich et al., 2019), a thorough risk-benefit assessment for off-label use of ADHD medications is pivotal (Leporini et al., 2022).

It has been demonstrated that methylphenidate elevates the concentration of dopamine within the nigrostriatal pathway, thereby intensifying the symptoms of tic disorder (Bailey, 2003). As a result, clinicians have displayed reluctance to prescribe stimulants for the treatment of children presenting with both ADHD and tics, due to the potential aggravation of tic symptoms. Our investigation substantiates these concerns, as we uncovered noteworthy adverse reaction signals for tics among patients utilizing methylphenidate (0–5 years: PRR = 16.66; 6–12 years: PRR = 5.64; 13–18 years: PRR = 3.19; ≥19 years: PRR = 31.79). Among amphetamine users, we also found adverse signals about tic (6–12 years: PRR = 9.96; 13–18 years: PRR = 11.41; ≥19 years: PRR = 25.81). Upon further stratification by gender, we found that the strength of adverse reaction signals was roughly the same for both males and females. Osland et al. (2018) stated that, in certain instances, the stimulant medication methylphenidate or amphetamine could exacerbate tics; therefore, they suggested the use of atomoxetine as a potential alternative therapy. Nevertheless, it is crucial to acknowledge that we also identified adverse reaction signals for tics in patients who received atomoxetine (6–12 years: PRR = 7.16; 13–18 years: PRR = 12.59; ≥19 years: PRR = 39.42). Consequently, atomoxetine, methylphenidate, and amphetamine may provoke or worsen tic manifestations in a limited number of patients, particularly among boys and those with a prior history of tics (Yang et al., 2017). Hence, it is imperative for physicians to remain cognizant of and vigilant towards this potential complication.

### 4.4 Limitations

This study has certain limitations stemming from the FAERS database and the study design. Firstly, the FDA does not require proof of a causal relationship between the adverse event and the drug at the time of the report submission, which prevents us from establishing a causal relationship between the occurrence of adverse reactions and drug use, or determining whether the adverse reactions are attributable to the drugs, ADHD comorbidities, or other factors. Secondly, the FDA cannot collect all reports on adverse events or medication errors for a drug product. The ability to report adverse events or medication errors is influenced by several factors, such as when the product was marketed and the level of public awareness of adverse events and medication errors. FAERS data cannot be used to calculate the incidence of adverse events or medication errors in the monitored population, and are primarily used for hypothesis generation rather than confirmation. Detailed information from clinical follow-ups and other studies would be required to verify the potential associations identified in our analysis. Finally, due to the accessibility of medications in different regions of the world, this study focused only on the most commonly used medications in ADHD treatment rather than all medications.

## 5 Conclusion

In conclusion, our pharmacovigilance analysis has revealed significant variations in the safety profiles of methylphenidate, atomoxetine, and amphetamine across different age groups and genders. We discovered prominent safety signals, with those associated with coronary artery dissection induced by methylphenidate and amphetamine, as well as those linked to suicide, demanding particular attention. These findings underscore the importance of personalized prescribing and careful monitoring of patients taking these medications. However, the limitations of this study, including potential inaccuracies and underreporting in the FAERS database and the inability to establish causality, highlight the need for further research. We recommend in-depth, prospective studies to confirm these findings and explore the mechanisms underlying these adverse reactions. Meanwhile, clinicians should be aware of these potential risks and consider them in their decision-making process, especially for patients who are at higher risk. Patient education about these potential adverse reactions and regular monitoring should be a standard part of the treatment plan.

## Data Availability

The raw data supporting the conclusion of this article will be made available by the authors, without undue reservation.

## References

[B2] Anders T. Sharfstein S. (2006). ADHD drugs and cardiovascular risk. N. Engl. J. Med. 354 (21), 2296–2298. 10.1056/NEJMc061187 16723628

[B3] Anderson K. N. Ailes E. C. Danielson M. Lind J. N. Farr S. L. Broussard C. S. (2018). Attention-deficit/hyperactivity disorder medication prescription claims among privately insured women aged 15-44 Years - United States, 2003-2015. MMWR Morb. Mortal. Wkly. Rep. 67 (2), 66–70. 10.15585/mmwr.mm6702a3 29346342 PMC5772805

[B4] Arun P. Sahni S. (2014). Methylphenidate and suicidal ideation: report of two cases. Indian J. Psychiatry 56 (1), 79–81. 10.4103/0019-5545.124721 24574564 PMC3927251

[B5] Bailey K. P. (2003). Pharmacological treatments for ADHD and the novel agent atomoxetine. J. Psychosoc. Nurs. Ment. Health Serv. 41 (8), 12–17. 10.3928/0279-3695-20030801-09 13677007

[B6] Ballard J. E. Boileau R. A. Sleator E. K. Massey B. H. Sprague R. L. (1976). Cardiovascular responses of hyperactive children to methylphenidate. Jama 236 (25), 2870–2874. 10.1001/jama.1976.03270260026021 792490

[B7] Biederman J. Spencer T. J. Monuteaux M. C. Faraone S. V. (2010). A naturalistic 10-year prospective study of height and weight in children with attention-deficit hyperactivity disorder grown up: sex and treatment effects. J. Pediatr. 157 (4), 635–640. 10.1016/j.jpeds.2010.04.025 20605163 PMC2943875

[B8] Blake K. V. Zaccaria C. Domergue F. La Mache E. Saint-Raymond A. Hidalgo-Simon A. (2014). Comparison between paediatric and adult suspected adverse drug reactions reported to the European medicines agency: implications for pharmacovigilance. Paediatr. Drugs 16 (4), 309–319. 10.1007/s40272-014-0076-2 24898717

[B9] Böhm R. Bulin C. Waetzig V. Cascorbi I. Klein H. J. Herdegen T. (2021). Pharmacovigilance-based drug repurposing: the search for inverse signals via OpenVigil identifies putative drugs against viral respiratory infections. Br. J. Clin. Pharmacol. 87 (11), 4421–4431. 10.1111/bcp.14868 33871897

[B10] Bracken L. Nunn A. Peak M. Turner M. (2018). Challenges in the assessment of adverse drug reactions in children and neonates. Adverse Drug React. Bull. 308 (1), 1191–1194. 10.1097/fad.0000000000000030

[B11] Brown E. G. Wood L. Wood S. (1999). The medical dictionary for regulatory activities (MedDRA). Drug Saf. 20 (2), 109–117. 10.2165/00002018-199920020-00002 10082069

[B12] Buchhorn R. Müller C. Willaschek C. Norozi K. (2012). How to predict the impact of methylphenidate on cardiovascular risk in children with attention deficit disorder: methylphenidate improves autonomic dysfunction in children with ADHD. ISRN Pharmacol. 2012, 170935. 10.5402/2012/170935 22530135 PMC3316982

[B13] Capuano A. Scavone C. Rafaniello C. Arcieri R. Rossi F. Panei P. (2014). Atomoxetine in the treatment of attention deficit hyperactivity disorder and suicidal ideation. Expert Opin. Drug Saf. 13 (1), S69–S78. 10.1517/14740338.2014.941804 25171160

[B14] Chang Z. Quinn P. D. O'Reilly L. Sjölander A. Hur K. Gibbons R. (2020). Medication for attention-deficit/hyperactivity disorder and risk for suicide attempts. Biol. Psychiatry 88 (6), 452–458. 10.1016/j.biopsych.2019.12.003 31987492 PMC7292769

[B15] Clavenna A. Bonati M. (2017). Pediatric pharmacoepidemiology - safety and effectiveness of medicines for ADHD. Expert Opin. Drug Saf. 16 (12), 1335–1345. 10.1080/14740338.2017.1389894 28984477

[B16] Cortese S. Holtmann M. Banaschewski T. Buitelaar J. Coghill D. Danckaerts M. (2013). Practitioner review: current best practice in the management of adverse events during treatment with ADHD medications in children and adolescents. J. Child. Psychol. Psychiatry 54 (3), 227–246. 10.1111/jcpp.12036 23294014

[B17] Dagenais S. Scranton R. Joyce A. R. Vick C. C. (2018). A comparison of approaches to identify possible cases of local anesthetic systemic toxicity in the FDA Adverse Event Reporting System (FAERS) database. Expert Opin. Drug Saf. 17 (6), 545–552. 10.1080/14740338.2018.1474200 29745266

[B18] Danielson M. L. Visser S. N. Chronis-Tuscano A. DuPaul G. J. (2018). A national description of treatment among United States children and adolescents with attention-deficit/hyperactivity disorder. J. Pediatr. 192, 240–246. 10.1016/j.jpeds.2017.08.040 29132817 PMC5732840

[B19] Evans S. J. Waller P. C. Davis S. (2001). Use of proportional reporting ratios (PRRs) for signal generation from spontaneous adverse drug reaction reports. Pharmacoepidemiol Drug Saf. 10 (6), 483–486. 10.1002/pds.677 11828828

[B20] Faraone S. V. Biederman J. Morley C. P. Spencer T. J. (2008). Effect of stimulants on height and weight: a review of the literature. J. Am. Acad. Child. Adolesc. Psychiatry 47 (9), 994–1009. 10.1097/CHI.ObO13e31817eOea7 18580502

[B21] Fettahoglu E. C. Satilmis A. Gokcen C. Ozatalay E. (2009). Oral megadose methylphenidate ingestion for suicide attempt. Pediatr. Int. 51 (6), 844–845. 10.1111/j.1442-200X.2009.02929.x 20158630

[B22] Franconi F. Campesi I. (2014). Sex and gender influences on pharmacological response: an overview. Expert Rev. Clin. Pharmacol. 7 (4), 469–485. 10.1586/17512433.2014.922866 24857340

[B23] Fukazawa C. Hinomura Y. Kaneko M. Narukawa M. (2018). Significance of data mining in routine signal detection: analysis based on the safety signals identified by the FDA. Pharmacoepidemiol Drug Saf. 27 (12), 1402–1408. 10.1002/pds.4672 30324671

[B25] Hanć T. Cieślik J. (2008). Growth in stimulant-naive children with attention-deficit/hyperactivity disorder using cross-sectional and longitudinal approaches. Pediatrics 121 (4), e967–e974. 10.1542/peds.2007-1532 18381524

[B26] Harpaz R. DuMouchel W. LePendu P. Bauer-Mehren A. Ryan P. Shah N. H. (2013). Performance of pharmacovigilance signal-detection algorithms for the FDA adverse event reporting system. Clin. Pharmacol. Ther. 93 (6), 539–546. 10.1038/clpt.2013.24 23571771 PMC3857139

[B27] Holm L. Ekman E. Jorsäter Blomgren K. (2017). Influence of age, sex and seriousness on reporting of adverse drug reactions in Sweden. Pharmacoepidemiol Drug Saf. 26 (3), 335–343. 10.1002/pds.4155 28071845

[B28] Kendall T. Taylor E. Perez A. Taylor C. Guideline Development Group (2008). Diagnosis and management of attention-deficit/hyperactivity disorder in children, young people, and adults: summary of NICE guidance. Bmj 337, a1239. 10.1136/bmj.a1239 18815170

[B29] Kim E. S. H. (2020). Spontaneous coronary-artery dissection. N. Engl. J. Med. 383 (24), 2358–2370. 10.1056/NEJMra2001524 33296561

[B30] Kok F. M. Groen Y. Fuermaier A. B. M. Tucha O. (2020). The female side of pharmacotherapy for ADHD-A systematic literature review. PLoS One 15 (9), e0239257. 10.1371/journal.pone.0239257 32946507 PMC7500607

[B31] Kooij S. J. Bejerot S. Blackwell A. Caci H. Casas-Brugué M. Carpentier P. J. (2010). European consensus statement on diagnosis and treatment of adult ADHD: the European Network Adult ADHD. BMC Psychiatry 10, 67. 10.1186/1471-244x-10-67 20815868 PMC2942810

[B32] Kramer J. R. Loney J. Ponto L. B. Roberts M. A. Grossman S. (2000). Predictors of adult height and weight in boys treated with methylphenidate for childhood behavior problems. J. Am. Acad. Child. Adolesc. Psychiatry 39 (4), 517–524. 10.1097/00004583-200004000-00022 10761355

[B33] Kratochvil C. J. (2012). ADHD pharmacotherapy: rates of stimulant use and cardiovascular risk. Am. J. Psychiatry 169 (2), 112–114. 10.1176/appi.ajp.2011.11111703 22318789

[B34] Kratochvil C. J. Wilens T. E. Greenhill L. L. Gao H. Baker K. D. Feldman P. D. (2006). Effects of long-term atomoxetine treatment for young children with attention-deficit/hyperactivity disorder. J. Am. Acad. Child. Adolesc. Psychiatry 45 (8), 919–927. 10.1097/01.chi.0000222788.34229.68 16865034

[B35] Lamberti M. Italiano D. Guerriero L. D'Amico G. Siracusano R. Ingrassia M. (2015). Evaluation of acute cardiovascular effects of immediate-release methylphenidate in children and adolescents with attention-deficit hyperactivity disorder. Neuropsychiatr. Dis. Treat. 11, 1169–1174. 10.2147/ndt.S79866 26056451 PMC4431494

[B36] Lee W. C. Chang H. M. Huang M. C. Pan C. H. Su S. S. Tsai S. Y. (2021). All-cause and suicide mortality among people with methamphetamine use disorder: a nation-wide cohort study in taiwan. Addiction 116 (11), 3127–3138. 10.1111/add.15501 33788344

[B37] Leporini C. De Sarro C. Palleria C. Caccavo I. Piro B. Citraro R. (2022). Pediatric drug safety surveillance: a 10-year analysis of adverse drug reaction reporting data in calabria, southern Italy. Drug Saf. 45 (11), 1381–1402. 10.1007/s40264-022-01232-w 36112324 PMC9483327

[B38] Liang E. F. Lim S. Z. Tam W. W. Ho C. S. Zhang M. W. McIntyre R. S. (2018). The effect of methylphenidate and atomoxetine on heart rate and systolic blood pressure in young people and adults with attention-deficit hyperactivity disorder (ADHD): systematic review, meta-analysis, and meta-regression. Int. J. Environ. Res. Public Health 15 (8), 1789. 10.3390/ijerph15081789 30127314 PMC6121294

[B39] Mangoni A. A. Jackson S. H. (2004). Age-related changes in pharmacokinetics and pharmacodynamics: basic principles and practical applications. Br. J. Clin. Pharmacol. 57 (1), 6–14. 10.1046/j.1365-2125.2003.02007.x 14678335 PMC1884408

[B40] Martinez-Raga J. Knecht C. Szerman N. Martinez M. I. (2013). Risk of serious cardiovascular problems with medications for attention-deficit hyperactivity disorder. CNS Drugs 27 (1), 15–30. 10.1007/s40263-012-0019-9 23160939

[B41] Maxwell R. A. Plummer A. J. Ross S. D. Daniel A. I. (1958). Studies concerning the cardiovascular actions of the central nervous stimulant, methylphenidate. J. Pharmacol. Exp. Ther. 123 (1), 22–27. 13539785

[B42] Merikangas K. R. He J. P. Brody D. Fisher P. W. Bourdon K. Koretz D. S. (2010). Prevalence and treatment of mental disorders among US children in the 2001-2004 NHANES. Pediatrics 125 (1), 75–81. 10.1542/peds.2008-2598 20008426 PMC2938794

[B43] Öhlund L. Ott M. Lundqvist R. Sandlund M. Salander Renberg E. Werneke U. (2020). Suicidal and non-suicidal self-injurious behaviour in patients with bipolar disorder and comorbid attention deficit hyperactivity disorder after initiation of central stimulant treatment: a mirror-image study based on the LiSIE retrospective cohort. Ther. Adv. Psychopharmacol. 10, 2045125320947502. 10.1177/2045125320947502 32843959 PMC7418477

[B44] Osland S. T. Steeves T. D. Pringsheim T. (2018). Pharmacological treatment for attention deficit hyperactivity disorder (ADHD) in children with comorbid tic disorders. Cochrane Database Syst. Rev. 6 (6), Cd007990. 10.1002/14651858.CD007990.pub3 29944175 PMC6513283

[B45] Peyre H. Hoertel N. Cortese S. Acquaviva E. Limosin F. Delorme R. (2013). Long-term effects of ADHD medication on adult height: results from the NESARC. J. Clin. Psychiatry 74 (11), 1123–1124. 10.4088/JCP.13l08580 24330902

[B46] Poulton A. (2005). Growth on stimulant medication; clarifying the confusion: a review. Arch. Dis. Child. 90 (8), 801–806. 10.1136/adc.2004.056952 16040876 PMC1720538

[B47] Purper-Ouakil D. Ramoz N. Lepagnol-Bestel A. M. Gorwood P. Simonneau M. (2011). Neurobiology of attention deficit/hyperactivity disorder. Pediatr. Res. 69 (5), 69R–76r. 10.1203/PDR.0b013e318212b40f 21289544

[B48] Roden D. M. (2004). Drug-induced prolongation of the QT interval. N. Engl. J. Med. 350 (10), 1013–1022. 10.1056/NEJMra032426 14999113

[B49] Rowland A. S. Skipper B. J. Umbach D. M. Rabiner D. L. Campbell R. A. Naftel A. J. (2015). The prevalence of ADHD in a population-based sample. J. Atten. Disord. 19 (9), 741–754. 10.1177/1087054713513799 24336124 PMC4058092

[B50] Safer D. J. (2011). Age-grouped differences in adverse drug events from psychotropic medication. J. Child. Adolesc. Psychopharmacol. 21 (4), 299–309. 10.1089/cap.2010.0152 21851188

[B52] Spencer T. Biederman J. Wright V. Danon M. (1992). Growth deficits in children treated with desipramine: a controlled study. J. Am. Acad. Child. Adolesc. Psychiatry 31 (2), 235–243. 10.1097/00004583-199203000-00009 1564024

[B53] Stammschulte T. Pitzer M. Rascher W. Becker M. Pohlmann U. Ostermayer S. (2022). Acute myocardial infarction due to spontaneous coronary artery dissection in a 6-year-old boy with ADHD on the third day of treatment with methylphenidate. Eur. Child. Adolesc. Psychiatry 31 (6), 939–945. 10.1007/s00787-021-01729-2 33537905

[B54] Stein M. A. (2008). Impairment associated with adult ADHD. CNS Spectr. 13 (8), 9–11. 10.1017/s1092852900003187 18704030

[B55] Stolarz A. J. Rusch N. J. (2015). Gender differences in cardiovascular drugs. Cardiovasc Drugs Ther. 29 (4), 403–410. 10.1007/s10557-015-6611-8 26227895

[B56] Stricker B. Cheung K. Verhamme K. (2022). General practice database on mortality in adults on methylphenidate: cohort study. BMJ Open 12 (8), e057303. 10.1136/bmjopen-2021-057303 PMC9422798 36028269

[B57] Swanson J. M. Elliott G. R. Greenhill L. L. Wigal T. Arnold L. E. Vitiello B. (2007). Effects of stimulant medication on growth rates across 3 years in the MTA follow-up. J. Am. Acad. Child. Adolesc. Psychiatry 46 (8), 1015–1027. 10.1097/chi.0b013e3180686d7e 17667480

[B59] Tobaiqy M. Stewart D. Helms P. J. Williams J. Crum J. Steer C. (2011). Parental reporting of adverse drug reactions associated with attention-deficit hyperactivity disorder (ADHD) medications in children attending specialist paediatric clinics in the UK. Drug Saf. 34 (3), 211–219. 10.2165/11586050-000000000-00000 21332245

[B60] Volkow N. D. Wang G. J. Fowler J. S. Molina P. E. Logan J. Gatley S. J. (2003). Cardiovascular effects of methylphenidate in humans are associated with increases of dopamine in brain and of epinephrine in plasma. Psychopharmacol. Berl. 166 (3), 264–270. 10.1007/s00213-002-1340-7 12589522

[B61] Wolraich M. Brown L. Brown R. T. DuPaul G. Earls M. Feldman H. M. (2011). Adhd: clinical practice guideline for the diagnosis, evaluation, and treatment of attention-deficit/hyperactivity disorder in children and adolescents. Pediatrics 128 (5), 1007–1022. 10.1542/peds.2011-2654 22003063 PMC4500647

[B62] Wolraich M. L. Hagan J. F. Jr. Allan C. Chan E. Davison D. Earls M. (2019). Clinical practice guideline for the diagnosis, evaluation, and treatment of attention-deficit/hyperactivity disorder in children and adolescents. Pediatrics 144 (4), e20192528. 10.1542/peds.2019-2528 31570648 PMC7067282

[B63] Wolraich M. L. McKeown R. E. Visser S. N. Bard D. Cuffe S. Neas B. (2014). The prevalence of ADHD: its diagnosis and treatment in four school districts across two states. J. Atten. Disord. 18 (7), 563–575. 10.1177/1087054712453169 22956714

[B64] Wonnacott D. Berringer R. (2016). Spontaneous coronary artery dissection: case report and review of the literature. Can. Fam. Physician 62 (12), 994–996. 27965334 PMC5154649

[B65] Xu G. Strathearn L. Liu B. Yang B. Bao W. (2018). Twenty-Year trends in diagnosed attention-deficit/hyperactivity disorder among US children and adolescents, 1997-2016. JAMA Netw. Open 1 (4), e181471. 10.1001/jamanetworkopen.2018.1471 30646132 PMC6324288

[B66] Yang R. Li R. Gao W. Zhao Z. (2017). Tic symptoms induced by atomoxetine in treatment of ADHD: a case report and literature review. J. Dev. Behav. Pediatr. 38 (2), 151–154. 10.1097/dbp.0000000000000371 27922902

[B67] Zablotsky B. Black L. I. Maenner M. J. Schieve L. A. Danielson M. L. Bitsko R. H. (2019). Prevalence and trends of developmental disabilities among children in the United States: 2009-2017. Pediatrics 144 (4), e20190811. 10.1542/peds.2019-0811 31558576 PMC7076808

